# miR-337 regulates the proliferation and invasion in pancreatic ductal adenocarcinoma by targeting HOXB7

**DOI:** 10.1186/s13000-014-0171-2

**Published:** 2014-09-03

**Authors:** Rui Zhang, Hong Leng, Junwen Huang, Yuwen Du, Yuanyuan Wang, Wenqiao Zang, Xiaonan Chen, Guoqiang Zhao

**Affiliations:** Department of emergency, the First Affiliated Hospital of Zhengzhou University, No.1 Jianshe Road, Zhengzhou, Henan 450052 China; Department of immunology and pathogen biology, Luoyang Vocational & Technical College, Luoyang, Henan, 471000, China; College of Basic Medical Sciences, Zhengzhou University, No.100 Kexue Road, Zhengzhou, 450001 China

**Keywords:** Pancreatic ductal adenocarcinoma (PDAC), HOXB7, miR-337

## Abstract

**Background:**

miRNAs are involved in coordinating a variety of cellular processes by regulating their target genes. Aberrant expression of miRNAs is correlated with various cancers. Previous studies have shown that miR-337 is significantly down-regulated in pancreatic ductal adenocarcinoma (PDAC) and that its expression is negatively correlated to the expression of HOXB7. Both miR-337 and HOXB7 are associated with the prognosis of PDAC patients. The purpose of this study was to identify the molecular mechanisms by which miR-337 acts as a tumor suppressor in PDAC.

**Methods:**

Synthetic miR-337 mimics were transfected into PANC-1 and As-PC-1 cells using Lipofectamine™ 2000. The expression of HOXB7 protein was analyzed by Western blot. Luciferase reporter plasmids were constructed to confirm that HOXB7 3′UTR was the target of miR-337. The effect of miR-337 on cell proliferation was evaluated by CCK8 assay and colony formation assay, and cell invasion was evaluated by wound healing assay and transwell assay.

**Results:**

Western blot and luciferase activity assays identified HOXB7 as the target of miR-337. A CCK-8 assay showed the absorbance of cells transfected with miR-337 mimics to be less than that of control cells, and that the number of cell clones was significantly decreased by miR-337 expression. A wound healing assay showed the invasion rate of cells transfected with miR-337 mimics at 36 h to be markedly lower than in controls. The average number of cells penetrating the Matrigel was significantly lower than the controls.

**Conclusion:**

These findings suggest that miR-337 targets HOXB7 and effects significant suppression of PDAC cell proliferation and invasion.

**Virtual Slides:**

The virtual slide(s) for this article can be found here: http://www.diagnosticpathology.diagnomx.eu/vs/13000_2014_171

## Background

microRNAs (miRNAs) are small non-coding, cellular RNAs (17–27 bp) that function as sequence-specific regulators of gene expression through translational repression and transcript cleavage [1,2]. The discovery of miRNAs and their mode of action revealed an entirely new level of gene regulation. Many studies have shown that miRNAs play key roles in cellular differentiation, proliferation, and apoptosis [3–6]. Aberrant expression of miRNAs has been found to be related to various human diseases, including cancers [7–9]. In cancerous tissues, including pancreatic cancer tissues, microRNAs appear to be dysregulated such that those with tumor-suppressor activity are abrogated [10,11]. Those that are overexpressed may function as oncogenes promoting proliferation, migration and invasion and repressing apoptosis [12,13].

HOX genes belong to the superfamily of homeobox genes and encode transcriptional factors that regulate the expression of many downstream target genes.

Many studies have shown HOX genes to be highly expressed in many cancers, such as breast cancer [14], ovarian cancer [15], hepatocellular cancer [16], and leukemia [17]. In addition, aberrant expression of HOX genes was observed in pancreatic cancer [18]. During normal development, HOXB7 stimulates the proliferation and survival of progenitor cells. However, the over-expression of HOXB7 can markedly promote the transformation, proliferation, and survival of tumor cells [19,20].

In previous studies, high levels of HOXB7 expression and low levels of miR-337 were detected in human pancreatic ductal adenocarcinoma tissues (PDAC). The level of HOXB7 mRNA in cancer was found to be negatively correlated with the level of miR-337 [21]. The miRNA miR-337 may repress tumor cell proliferation and metastasis by targeting HOXB7. For this reason, the role of miR-337 on HOXB7 expression and the effects of miR-337 on proliferation and invasion in human pancreatic cancer cells in vitro were here examined.

## Materials and methods

### Cell culture

The human pancreatic cancer cell lines PANC-1 and As-PC-1 were purchased from the American Type Culture Collection. The cells were maintained in DMEM media supplemented with 10% fetal bovine serum (Gibco), 100 U/ml penicillin G (Invitrogen), and 0.1 mg/ml streptomycin sulfate (Invitrogen) at 37°C in a humidified, 5% CO_2_, 95% air atmosphere. This study was approved by the Research Ethics Committee of Zhengzhou University, China.

### RNA oligoribonucleotides and cell transfection

miR-337 mimics and the negative control (NC) were chemically synthesized by Shanghai GenePharma Co. Ltd. PANC- 1 and As-PC-1 cells were seeded in 6-well plates. A final concentration of either 50 nM RNA mimics or NC RNA was transfected into the cells using Lipofectamine™ 2000 (Invitrogen) according to the manufacturer’s protocol.

### Luciferase activity assay

The wild-type HOXB7 3′UTR luciferase reporter vector (pmirGLO-HOXB7-WT) was constructed by amplifying the human genomic DNA and then cloning it into the Xbal site of pmirGLO-control vector. The primers used were as follows: 5´ GGGATGGAGAAAGGGCAGAGGAAGA 3´ (foward), 5´ GCTACAGAACAGGTAGATAATATCC 3´ (reverse). The mutant type HOXB7 3′UTR luciferase reporter vector (pmirGLO-HOXB7-MUT) was established using a site-directed mutagenesis kit (Promega) with pmirGLO-HOXB7-Wt serving as a template. miR-337 mimics or NC (final concentration, 50 nM), 100 ng luciferase reporter plasmid were cotransfected into PANC-1 (or As-PC-1) cells of 90% confluence in 24-well plates. After 24 h, cells were lysed and luciferase activity was measured using a Dual-Luciferase Reporter Assay System (Promega).

### Western blot

The cultured cells were lysed by using RIPA buffer. BCA Protein Assay Kit was used to measure the protein concentrations. The total protein was loaded onto 10% SDS-PAGE gels and transferred to PVDF membranes. Membranes were probed overnight at 4°C with antibody recognizing HOXB7 (1:600, Santa Cruz), or GAPDH (1:2000, Santa Cruz) in TBST containing 1% BSA (w/v). Blots were then incubated for 2 h with anti-mouse secondary antibodies. Immune complexes were detected using an ECL Plus Detection Kit (Pierce, Rockford, IL, U.S.) and quantified using a scanning densitometer with molecular analysis software (Bio-Rad, Hercules, CA, U.S.).

### RNA extraction and qRT-PCR

Total RNA was isolated from cell lines using Trizol (Invitrogen). The expression levels of mature miR-337 and its precursor were quantified using quantitative real-time PCR (qRT-PCR) using the TaqMan human microRNA assay kits (Qiagen). U6 snRNA served as an internal normalized reference. The relative expression ratio of miR-337 was presented as the fold change as normalized to an endogenous reference (U6 RNA) relative to the normal cell line.

### Cell counting kit-8 (CCK-8) assay

After transfection, cells were seeded in a 96-well plate at a density of 1 × 10^4^ cells per well and incubated for four days. In vitro cell proliferation was evaluated using a CCK-8 (Beyotime Inst Biotech, China) according to manufacturer’s instructions. The absorbance was determined at 450 nm wavelength with a reference wavelength of 630 nm. All experiments were performed in triplicate.

### Colony formation assay

Twenty-four hours after RNA transfection, cells were trypsinized and plated on 6-well plates (1000 cells/well) and cultured of 2 weeks. Then the colonies were captured with the Olympus SZX12 and QCapture Pro software. Cell colonies >0.1 mm in diameter were counted under a microscope.

### Wound healing assay

Cells (6 × 10^6^ per well) at 12 h post-transfection were seeded in six-well plates and allowed to adhere for 24 h. Confluent monolayer cells were scratched with a 200 μl pipette tip and then washed three times with 1 × PBS to clear cell debris and suspension cells. Fresh serum-free medium was added, and the cells were allowed to close the wound for 36 h. Photographs were taken at 0 and 36 h at the same position relative to the wound.

### Transwell invasion assay

The lower side and upper sides of separate polycarbonate membranes (8 μm pores) of a transwell (Costar, Lowell, MA, U.S.) were coated with Matrigel (80 μg/well) for the invasion assays. The cells were added to the upper chambers of a transwell; after incubation for 16 h at 37°C, the cells on the lower side were stained with Giemsa stain. The level of invasion was determined using a microscope at × 200 magnification. All experiments were performed in triplicate.

### Statistical analysis

Statistical treatment was performed using SPSS 16.0 software. One-way analysis of variance (ANOVA) and the *χ*^2^ test were used to analyze the significance between groups. Multiple comparisons between the experimental group and control groups were made using Tukey’s HSD when the probability for ANOVA was statistically significant. All data represent mean ± SD. The statistical significance was set at *P* <0.05.

## Results

### HOXB7 is the target of miR-337

**Figure 1:**
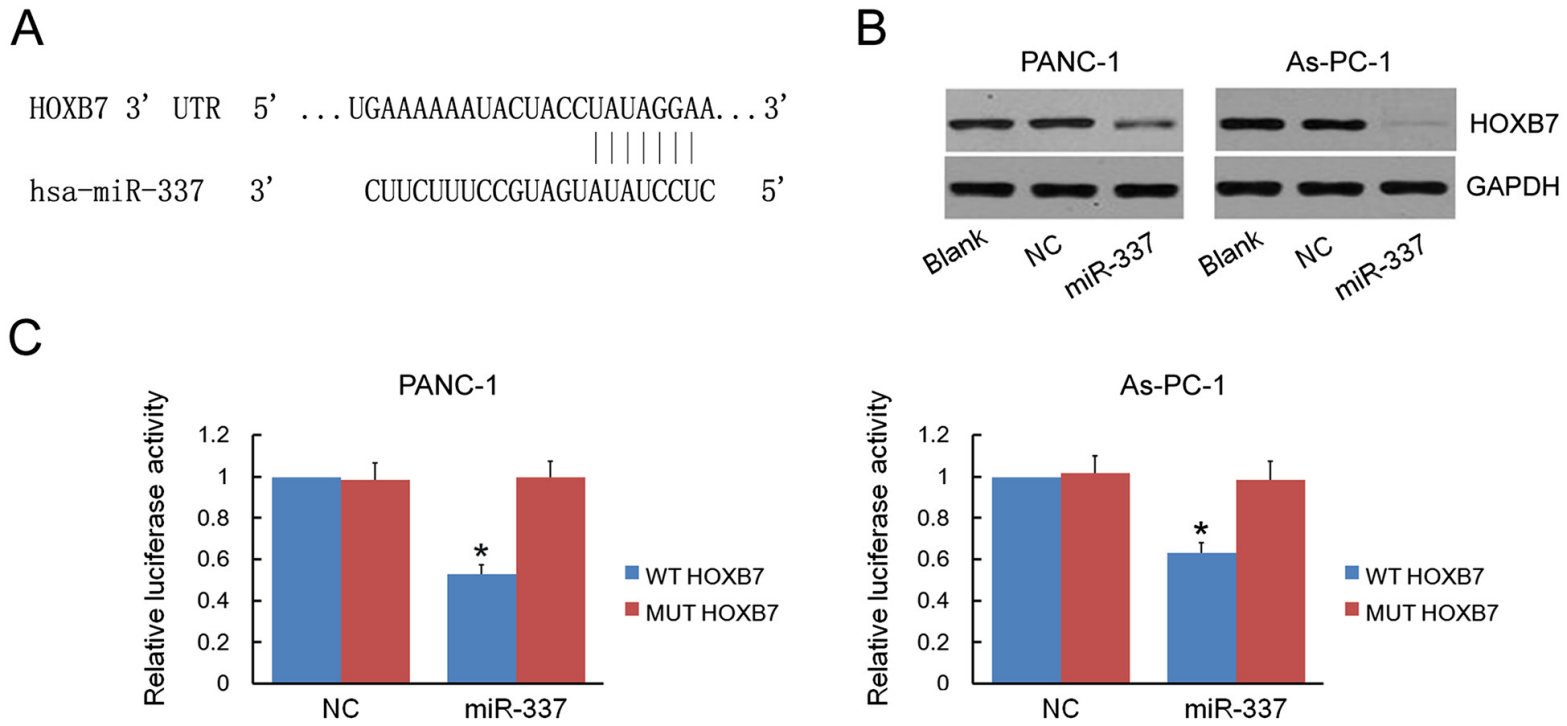
**HOXB7 is the target of miR-337. A)** Sequence alignment of miR-337 and its target sites in 3′UTR of HOXB7 was revealed by four prediction programs (miRBase, RNA22, miRanda, and TargetScan). **B)** Western blot was performed to detect the expression of HOXB7 protein in PANC-1 and AsPC-1. The levels of HOXB7 protein were lower in cells transfected with miR-337 mimics than in control cells (untransfected or transfected with NC). **C)** There was significantly less relative luciferase activity in cells cotransfected with HOXB7-3′UTR-WT and miR-337 mimics than in cells cotransfected with HOXB7-3′UTR-MUT and miR-337 mimics or in cells cotransfected with HOXB7-3′UTR-WT (or HOXB7-3′UTR-MUT) and NC. miR-337 mimics obviously inhibited luciferase activity in cells transfected with HOXB7-3′UTR-WT. Asterisks indicate significance at *P* <0.05.

Whether miR-337 affected the expression of HOXB7 protein was investigated by Western blot analysis. The levels of HOXB7 protein were clearly lower in miR-337 overexpressing cells (PANC-1 and As-PC-1) than in control cells (Figure 1B). Four prediction programs (miRBase, RNA22, miRanda, and TargetScan) were used to search for potential miR-337 targets to characterize the mechanism associated with miR-337 action. This analysis identified HOXB7 as a potential target of miR-337 (Figure 1A). In order to further support these results, two reporter plasmids carrying a wild-type HOXB7 3′UTR and a mutant HOXB7 3′UTR, respectively, were constructed. Cotransfection experiments showed that miR-337 mimics obviously inhibited luciferase activity in cells transfected with pmirGLO-HOXB7-WT but not in cells transfected with pmirGLO-HOXB7-MUT. The inhibitory effect was completely abrogated with pmirGLO-HOXB7-MUT, which contained a mutation in the miR-337 binding site (Figure 1C, D).

### miR-337 over-expression reduced tumor cell proliferation

**Figure 2:**
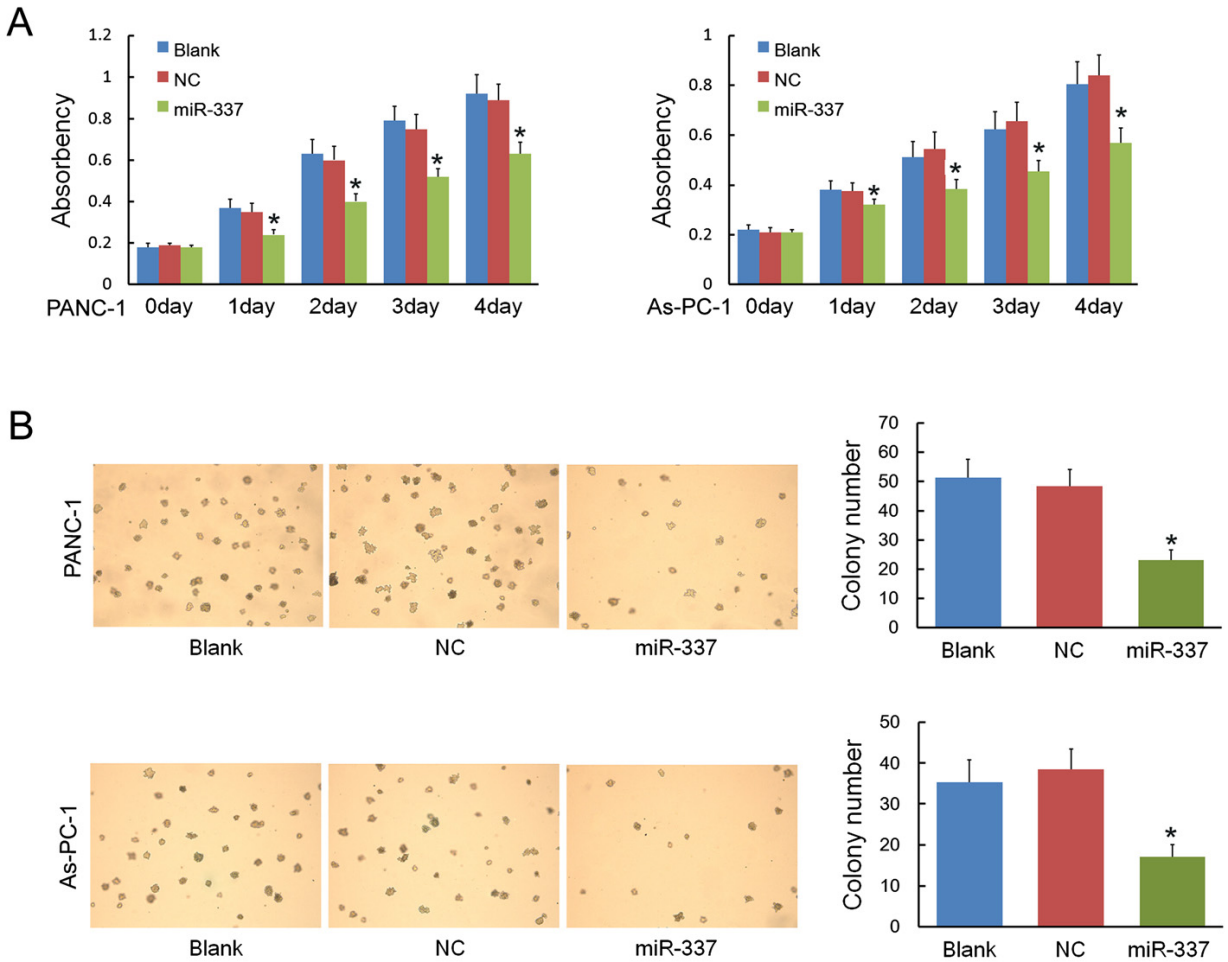
**miR-337 reduced cell proliferation in vitro.** The effects of miR-337 mimics (or NC) on the proliferation of PANC-1 and As-PC-1 cells in vitro were detected by CCK-8 assay and colony formation assay. **A)** After transfection with miR-337 mimics (or NC), PANC-1 and As-PC-1cells were seeded in a 96-well plate and incubated for four days. CCK-8 assay shows the absorbance of cells transfected with miR-337 mimics decreased compared to the control groups (NC and blank control) on days 0–4. **B)** Twenty-four hours after transfection, PANC-1 or As-PC-1cells were plated on 6-well plates and cultured of 2 weeks. A colony formation assay shows that cells transfected with miR-337 mimics formed significantly fewer colonies than the control groups (NC and blank control). Asterisks indicate significance at *P* < 0.05.

CCK-8 for proliferation assay was performed for PANC-1 and As-PC-1 cell lines, which were transfected with either miR-337 mimics or NC. Marked inhibition of growth was observed on the first day after transfection, and a marked inhibitory effect on cell proliferation lasted for four days (Figure 2A). The biological effect of miR-337 mimics on anchorage-independent colony formation in soft agar was also determined. After 2 weeks significantly less (*P* < 0.05) PANC-1 (or As-PC-1) cells colony forming potential was observed in cells treated with miR-337 mimics than in the blank and negative control groups, as assessed by the number of clones (Figure 2B).

### miR-337 over-expression reduced tumor cell invasion

**Figure 3:**
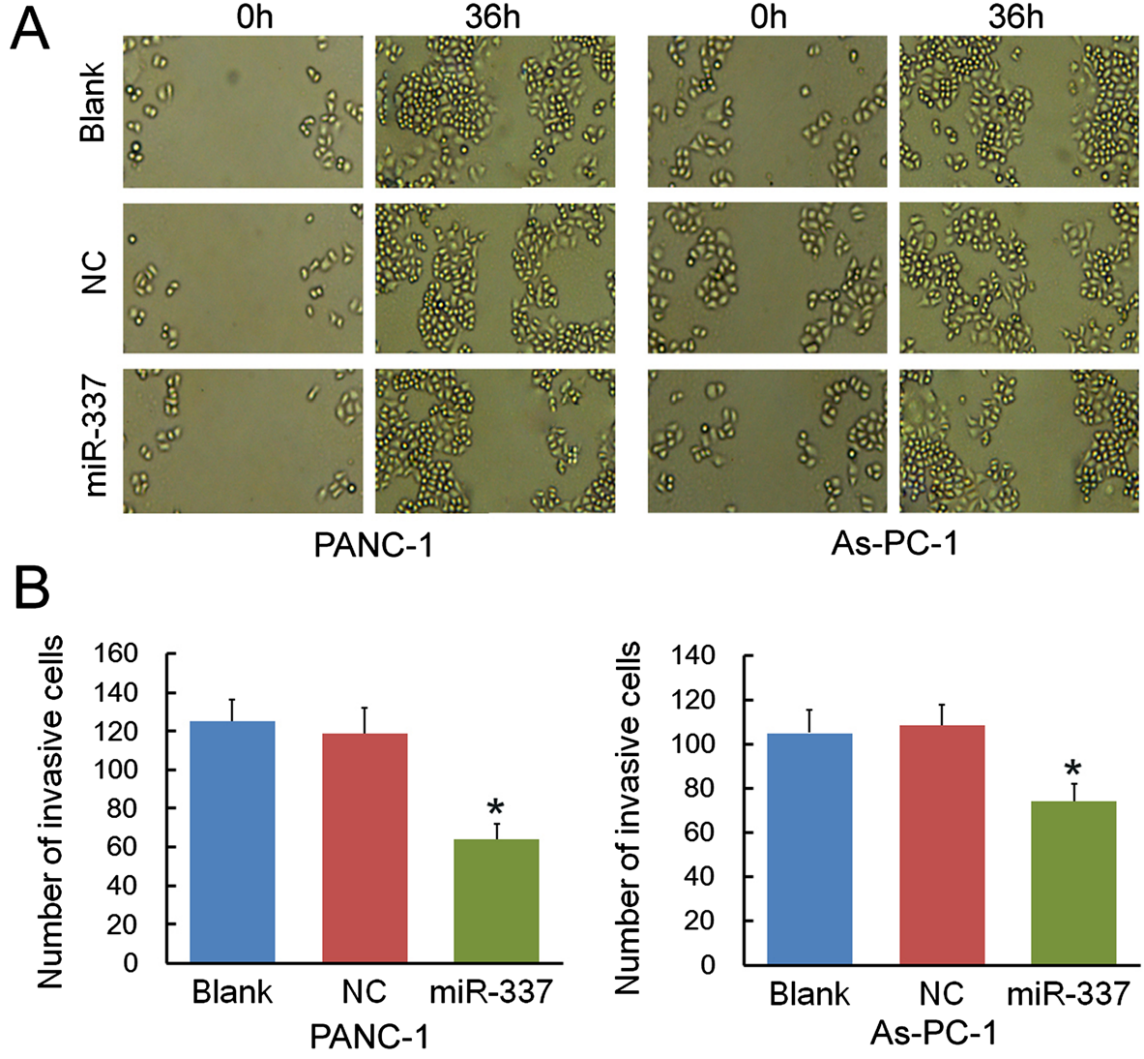
**miR-337 reduced cell invasion in vitro.** The effects of miR-337 mimics (or NC) on the proliferation of PANC-1 and As-PC-1 cells in vitro were detected by wound healing assay and transwell invasion assay. **A)** PANC-1 and As-PC-1 cells at 12 h post-transfection were seeded in six-well plates and cultured for 24 h. Confluent monolayer cells were scratched with a 200 μl pipette tip and were allowed to close the wound for 36 h. Wound healing assays show the rate of invasion of PANC-1 (or As-PC-1) cells transfected with miR-337 mimics at 36 h were markedly lower than in the control groups (blank control and cells transfected with miR-337 NC). **B)** The number of cells that migrate through the Matrigel into the lower surface of the polycarbonic membrane was determined in a transwell invasion assay. Cell invasion was significantly less pronounced in miR-337-mimic-treated cells than in the blank control and NC groups. Asterisks indicate significance at *P* < 0.05.

To evaluate the roles of miR-337 in the regulation of cell mobility, the invasion ability of PANC-1 (or As-PC-1) cells in vitro after different transfections was examined using a wound healing assay and transwell invasion assay. For a wound healing assay, PANC-1 (or As-PC-1) cells transfected with miR-337 mimics at 36 h after incubation exhibited a slower rate of invasion than that of miR-337 NC and untransfected group (Figure 3A). To further evaluate the impact of miR-337 over-expression on cell invasion, PANC-1 (or As-PC-1) cells were treated with the different oligonucleotides and placed in a transwell chamber. The numbers of miR-337-mimic-treated PANC-1 and As-PC-1 cells invading through the Matrigel were significantly lower (*P* < 0.05, *P* < 0.05) than in the blank control and the NC groups (Figure 3B). These results demonstrate that miR-337 over-expression inhibits the invasive ability of PANC-1 and As-PC-1cells in vitro.

## Discussion

Pancreatic cancer is one of the most common causes of cancer-related death. It is a global public health problem. Unfortunately, pancreatic cancer is highly aggressive, highly metastatic and currently extremely difficult to diagnose. Because there are no effective therapeutic agents, there is an urgent need to find and assess new ones.

HOX genes encode transcription factors that regulate many vital processes, such as differentiation, apoptosis, motility, angiogenesis, and receptor signaling. In adults, few HOX genes are constitutively expressed any tissues or organs, and most of them are silent [22,23]. Dysregulation of HOX gene expression is common in human cancers. It has been shown that overexpression of HOXB7 is involved in the differentiation, proliferation, and invasion of many cancer cells in vitro [24–26]. Furthermore, its overexpression was demonstrated to be closely related not only to the clinical invasive and aggressive characteristics, such as high Dukes stage, T stage, distant metastasis-positive tumors, and high proliferation index but also to poor prognosis of patients with tumors [19,20,27].

In humans, miRNAs play important roles in cellular physiology, development, and disease by negatively regulating gene expression through translational repression or post-transcriptional degradation [28]. miR-337 has been reported to be expressed abnormally in many cancers [29,30] such as breast cancer [31], esophageal squamous cell carcinoma [32], and lung cancer [33]. Its expression was found to be related to the survival of patients with ovarian cancer [34] and gastric cancer [35]. Previous studies showed that levels of miR-337 were low in human PDAC tissues, but levels of HOXB7 were high, and there was a negative correlation (*r* = −0.719, *P* < 0.01) between the level of HOXB7 mRNA and the level of miR-337. In addition, expression of both was related to TNM stage, lymph node status, and length of patient survival. These data suggest that miR-337 is crucial to the progression and metastasis of PDAC by regulating the expression of HOXB7.

To provide further evidence that miR-337contributes to the development of PDAC, two cancer cell lines, PANC-1 and As-PC-1, were used to perform gain-of-function experiments. PANC-1 cell line was started from a human pancreatic carcinoma of a ductal cell origin, while As-PC-1 cell line was established from the ascites of a patient with adenocarcinoma of the head of the pancreas. Morphologically the two pancreatic tumor epithelioid cell lines grow to confluency with moderately tight adhesion to the culture plastic surface, and they are suitable transfection hosts. PANC-1 and As-PC-1 were transfected with miR-337 mimics, and the expression of HOXB7 protein was inhibited using Western blot analysis. Bioinformatics analysis has also shown HOXB7 to be a potential target of miR-337.

miRNAs regulate their target genes through the 3′UTR of the gene. The 3′UTR of HOXB7 mRNA was linked to the downstream of luciferase gene in pMIR reporter plasmid. In cells over-expressing miR-337, transfection of wild HOXB7 luciferase reporter vector produced a significant decrease in luciferase reporter gene activity, but no reduction was observed in the case of the mutant luciferase reporter gene. The luciferase activity assay further confirmed that miR-337 regulates the expression of HOXB7 by targeting its 3′UTR.

Upregulated expression of miR-337 was shown to suppress cell proliferation, invasion, and metastasis in PANC-1 and As-PC-1. Results indicated that miR-337 could inhibit the proliferation of pancreatic cancer cells in both CCK-8 assay and in colony formation assay. Cell invasion is a significant aspect of cancer progression. It involves the migration of tumor cells into contiguous tissues and the dissolution of extracellular matrix proteins. The weakened invasive ability of cancer cells transfected with miR-337 mimics was observed in the wound healing and transwell invasion assays. These results are consistent with the data collected using clinical pancreatic cancer samples.

## Conclusions

In summary, these results demonstrated that overexpression of miR-337 can suppress cell proliferation, migration, and invasion in human pancreatic cancer cell lines by targeting HOXB7. These findings suggest that miR-337 may play an important role in the development and metastasis of PDAC. miR-337 may have therapeutic potential for controlling or preventing PDAC progression and metastasis.

## Consent

Written informed consent was obtained from the patient for the publication of this report and any accompanying images.

## Abbreviations

PDAC: Pancreatic ductal adenocarcinoma
HOXB7: Homeobox B7
